# Friendship fosters well-being, exclusion may hurt girls’ mathematics: a two-wave cross-lagged panel model of peer relations, affect, and achievement

**DOI:** 10.1007/s10212-026-01138-6

**Published:** 2026-05-30

**Authors:** Tomáš Lintner

**Affiliations:** The Mitchell Centre for Social Network Analysis, School of Social Sciences, The University of Manchester, Manchester, UK

**Keywords:** Early adolescence, Peer relations, Achievement, Well-being, Gender gap

## Abstract

This study investigates longitudinal associations between peer relations, affective well-being, and academic achievement during early adolescence. Data were drawn from a two-wave study of a large nationally stratified sample of Czech students transitioning from Grade 6 to Grade 7 (N = 2,609, ages 12–13). Cross-lagged panel models were used to examine reciprocal links between friendship, social exclusion, positive and negative affect, and achievement in mathematics and Czech language, assessed using item-response-theory-scaled tests. Friendship predicted increases in subsequent positive affect, whereas social exclusion predicted declines in positive affect. Exclusion was also associated with lower later achievement in Czech language across students and with reduced mathematics achievement specifically among girls, indicating domain- and gender-differentiated academic vulnerability. Affective well-being did generally predict achievement. These findings highlight the developmental importance of peer relations for emotional adjustment and suggest that experiences of exclusion may carry direct academic consequences, particularly for girls in mathematics.

**Supplementary Information:**

The online version contains supplementary material available at 10.1007/s10212-026-01138-6.

## Introduction

Adolescence represents a developmentally sensitive period characterized by rapid cognitive maturation, identity exploration, and heightened responsiveness to social evaluation (Blakemore & Mills, 2014; Steinberg, 2014). During this stage, peer relationships increasingly replace parental guidance as primary sources of emotional support, social comparison, and belonging (Brown & Larson, 2009). As young people spend substantial time within classroom peer contexts, their social experiences become closely intertwined with motivational processes, emotional adjustment, and academic engagement. Developmental and motivational theories consistently emphasize that the satisfaction of fundamental psychological needs for autonomy, competence, and relatedness is essential for adaptive functioning in educational settings. The self-system model (Connell & Wellborn, 1991) proposes that students’ engagement and learning depend on the degree to which social environments support these needs, while self-determination theory (Deci et al., 2017; Germani & Vespasiani, 2023) conceptualizes relatedness as a universal requirement for psychological flourishing and effective learning.

Peer relationships therefore gain particular developmental significance during the transition from childhood to adolescence, when students increasingly rely on classmates for emotional validation, identity construction, and normative guidance (Eccles & Roeser, 2011; Wentzel et al., 2021). Supportive friendships can enhance feelings of belonging, strengthen academic self-efficacy, and promote active participation in learning, whereas experiences of rejection or exclusion may undermine motivation and disrupt psychological functioning (Bukowski et al., 2011; Kiuru et al., 2020). Understanding how adolescents’ embeddedness within classroom peer networks contributes to emotional well-being and academic development thus represents a central challenge for contemporary educational research.

## Literature review

A substantial body of longitudinal research indicates that adolescents’ social integration within peer networks is closely associated with emotional adjustment. Positive peer functioning—including friendship quality, peer acceptance, and supportive interaction patterns—has been linked to higher psychological well-being and lower loneliness or distress (Chiu et al., 2021; Liu et al., 2024; Tikkanen et al., 2024). Supportive friendships may foster resilience by providing opportunities for mutual help, emotional validation, and shared coping with academic challenges (Ju et al., 2025). Conversely, social exclusion and marginalization are associated with increased negative affect, reduced self-worth, and elevated vulnerability to internalizing difficulties (Bukowski et al., 2011).

Beyond emotional consequences, peer relations also appear to play a role in academic functioning. Students who are socially integrated within classroom networks tend to demonstrate stronger motivation, greater engagement in learning activities, and more favorable academic trajectories (Kiuru et al., 2020; Shao et al., 2024). In contrast, marginalization within peer contexts has been linked to lower academic performance and increased likelihood of grade retention or downward educational mobility (Lubbers et al., 2006). Comparative and meta-analytic evidence further suggests moderate positive associations between peer acceptance and academic achievement across educational systems, highlighting peer relations as a key developmental resource alongside teacher and parental support (Wentzel et al., 2021; Yu et al., 2023).

One mechanism proposed to explain these associations involves emotional well-being. Positive affective states may enhance intrinsic motivation, attentional focus, and adaptive learning behaviors, whereas sustained negative affect may impair concentration and persistence (Scanlon et al., 2020; Schnell et al., 2025). Longitudinal findings indicate that subjective well-being can predict later academic outcomes, although evidence regarding directionality and causal ordering remains mixed (Kleinkorres et al., 2023; Tape et al., 2021; Yang et al., 2019). These inconsistencies suggest that emotional adjustment may represent one pathway linking peer experiences to achievement, while direct social influences on academic performance are also plausible.

Emerging research further indicates that the interplay between peer relations, emotional well-being, and academic development may be domain-specific and moderated by gender. Mathematics achievement, often embedded in processes of social comparison and gendered academic expectations, appears particularly sensitive to relational and affective dynamics within peer contexts. For instance, evidence suggests that girls’ mathematical confidence and performance can be shaped by perceived peer support and feelings of academic belonging (John et al., 2023), while stronger relational orientations in friendship networks may heighten emotional responsiveness to experiences of inclusion or exclusion (Rose & Rudolph, 2006). At the same time, large-scale syntheses indicate that gender differences in peer influence are contingent on contextual and domain-specific conditions rather than universal developmental patterns (Wentzel et al., 2021). Recent longitudinal evidence further highlights the importance of examining differentiated developmental pathways. Rémeau et al. (2025) observed an asymmetry in temporal associations between achievement and subjective school well-being: while well-being did not predict later academic performance, prior achievement showed domain-specific prospective effects on subsequent well-being. Higher numeracy in late primary school predicted increased subjective school well-being in adolescence, whereas higher literacy achievement was associated with subsequent declines in well-being. Notably, these longitudinal effects were concentrated among girls, with no comparable patterns observed for boys. Such findings underscore the importance of considering how academic domains intersect with social positioning, and how these processes may be moderated by gender.

## Present study

Despite extensive scholarship, several limitations characterize existing research. First, positive and negative peer experiences have often been examined separately, with studies focusing either on supportive friendships or on rejection and exclusion rather than modelling their simultaneous effects within a unified developmental framework. Second, although emotional well-being is frequently proposed as a mechanism linking peer relations and academic achievement, relatively few longitudinal investigations have explicitly disentangled direct and affect-mediated pathways. Third, potential gender- and domain-specific processes—such as heightened sensitivity of girls’ mathematics achievement to peer experiences—have rarely been tested using integrated structural models. Finally, much of the literature relies primarily on students’ subjective perceptions of peer relationships, whereas network-based indicators may provide complementary insights into the behavioral structure of social inclusion and marginalization within classroom contexts.

The present study addresses these gaps by examining longitudinal associations between peer relations, affective well-being, and academic achievement among a large nationally stratified cohort of Czech students transitioning from Grade 6 to Grade 7. Peer relations are conceptualized as objective indicators of social embeddedness derived from reciprocated friendship nominations and patterns of peer exclusion. Emotional well-being is operationalized in affective terms, distinguishing between positive and negative affect as proximal indicators of adolescents’ emotional adjustment. Academic achievement is assessed using item-response-theory-scaled tests in mathematics and Czech language, enabling domain-specific analysis of developmental trajectories.

To examine directional associations among these constructs, the study employs a cross-lagged panel modelling framework that simultaneously estimates reciprocal longitudinal pathways. This approach enables the investigation of whether peer relations prospectively predict changes in affective well-being and academic achievement, whether affective states predict subsequent academic outcomes, and whether these processes vary across gender and subject domains.

The study addresses the following research questions:

- **RQ1**: How are peer friendship and social exclusion prospectively associated with positive and negative affect, and with achievement in mathematics and Czech language?
- **RQ2**: To what extent do positive and negative affect predict subsequent academic achievement across subject domains?
- **RQ3**: Do gender and subject domain moderate longitudinal associations between peer relations, affective well-being, and academic achievement?

### Peer relations and affective well-being

Grounded in developmental and motivational theories, peer embeddedness is expected to shape adolescents’ emotional adjustment by fulfilling or frustrating the need for relatedness. Supportive friendships may enhance positive emotional experiences through social reinforcement and stress buffering, whereas exclusion may elicit negative affective reactions associated with loneliness and social threat.

- **H1a**: Higher levels of peer friendship will be associated with increases in positive affect.
- **H1b**: Higher levels of peer friendship will be associated with decreases in negative affect.
- **H1c**: Higher levels of peer exclusion will be associated with decreases in positive affect.
- **H1d**: Higher levels of peer exclusion will be associated with increases in negative affect.

### Peer relations and academic achievement

Beyond their emotional consequences, peer relationships may also influence academic functioning by shaping motivation, classroom participation, and perceived academic competence. Students who experience inclusion and support within peer networks may be more likely to engage in learning activities, whereas exclusion may undermine persistence and performance.

- **H2a**: Peer friendship will positively predict subsequent achievement in mathematics.
- **H2b**: Peer friendship will positively predict subsequent achievement in Czech language.
- **H2c**: Peer exclusion will negatively predict subsequent achievement in mathematics.
- **H2d**: Peer exclusion will negatively predict subsequent achievement in Czech language.

### Affective well-being and academic achievement

Emotional experiences are theorized to influence cognitive functioning and learning behaviors. Positive affect may facilitate attentional engagement and adaptive motivation, whereas sustained negative affect may interfere with concentration and academic persistence.

- **H3a**: Positive affect will positively predict subsequent achievement in mathematics.
- **H3b**: Positive affect will positively predict subsequent achievement in Czech language.
- **H3c**: Negative affect will negatively predict subsequent achievement in mathematics.
- **H3d**: Negative affect will negatively predict subsequent achievement in Czech language.

### Gender and domain moderation

Developmental and sociocultural perspectives suggest that peer and emotional processes may operate differently across genders and academic domains. In particular, girls’ achievement in mathematics may be more sensitive to experiences of belonging and peer support due to gendered expectations and relational orientations.

- **H4a**: Associations between peer relations and affective well-being will be stronger among girls than boys.
- **H4b**: Associations between peer relations and academic achievement will be stronger among girls than boys.
- **H4c**: Associations between affective well-being and academic achievement will be stronger among girls than boys.
- **H4d**: Combined effects of peer relations and affective well-being on achievement will be particularly pronounced for girls in mathematics.

## Methods

### Sample

Data were drawn from a nationally stratified multistage sample of Czech lower-secondary school students followed longitudinally from Grade 6 to Grade 7. Schools were selected using a stratified random sampling procedure based on administrative regions and the number of sixth-grade students enrolled in each school. Stratification quotas were determined using official statistics from the Czech Ministry of Education, Youth and Sports, ensuring coverage across major regional and school-size categories. Schools with fewer than 15 sixth-grade students were excluded from the sampling frame in order to ensure sufficient within-classroom participation.

Within each participating school, one sixth-grade classroom was randomly selected, and all students in the selected classroom were invited to participate. In schools offering both standard and extended instructional programs, two classrooms were randomly selected to capture variation in curricular tracks.

Participation rates varied across strata, with an average school-level response rate of approximately 18%, calculated according to the American Association for Public Opinion Research standards. Once a school participated, student participation within selected classes was high (95.2%). Because no cooperating school was obtained in one regional stratum (Liberec Region), the assessment of sample proportionality was conducted at the broader NUTS-2 regional level.

Table 1 compares the regional distribution of the analytic student sample with national population statistics for lower-secondary education. The comparison indicates that the achieved sample broadly reflects the regional structure of the Czech student population, although some deviations are present, including lower representation of students from Prague and modest overrepresentation of Northeast and Southeast regions. No post-stratification weights were applied in the analyses. Consequently, findings should be interpreted with consideration of potential participation-related selectivity.

**Table 1:** Sample in relation to full population regarding NUTS-2 region representation

NUTS-2 region	% in sample	% in Czech Republic
Southeast	17.9	15.8
Southwest	11.6	11.7
Moravian-Silesian	14.4	10.9
Prague	7.3	11.5
Northeast	16.4	14.5
Northwest	9.1	10.5
Central Moravia	10.8	10.9
Central Bohemia	12.3	14.2

The data were collected through an online survey administered by the Public Opinion Research Centre of the Institute of Sociology of the Czech Academy of Sciences. The first wave of data collection took place at the end of the 2022/23 school year, and the second wave was carried out at the end of the 2023/24 school year.

In total, the analytic sample comprised 2,609 students nested within 142 classrooms (M_size_ = 18.09, SD = 4.48, range = 6–29). As shown in Table 2, the sample included approximately equal proportions of girls and boys. Around 7% of students were of non-Czech origin, and the proportion of students whose parents held a higher-education degree was slightly lower than those without tertiary education. Across demographic variables, the proportion of missing data was approximately 13%.

**Table 2:** Sample characteristics

	*N*	%	missing *N*	missing %
gender			343	13.15
girls	1135	43.50		
boys	1131	43.35		
ethnicity			344	13.19
Czech	2085	79.92		
non-Czech	180	6.90		
parental education			354	13.57
non-HE	1282	49.14		
HE	973	37.29		

### Instruments

#### Peer relations

Two dimensions of peer relations were assessed: friendship integration and social exclusion. At both time points, students were presented with their full class roster and asked to nominate (a) classmates they considered friends, and (b) classmates with whom they would not want to share a desk. Both nomination tasks were unlimited, permitting nomination of any number of classmates.

Friendship integration was operationalized as the proportion of reciprocated friendship ties within the classroom. Specifically, for each student *i* in a classroom of *n* peers, a reciprocated friendship tie was counted when both student *i* nominated student *j* as a friend and student *j* nominated student *i* in return. The individual friendship score was then calculated as:

$${F}_{i}=\frac{{\sum}_{j\ne i}1\left[i\to j and j\to i\right]}{n-1}$$

where $1[\cdot ]$ is an indicator function equal to 1 when a mutual nomination existed between students *i* and *j*, and $n-1$ is the total number of classmates eligible for nomination. Reciprocated nominations were prioritized over unilateral ones because mutual recognition of a friendship tie constitutes a stronger indicator of genuine social integration and shared belonging within the peer network than one-sided nominations alone.

Social exclusion was operationalized as the proportion of classmates who nominated a given student as someone they would not want to share a desk with. For each student *i*, the exclusion score was calculated as:

$${E}_{i}=\frac{{\sum}_{j\ne i}1\left[ j\to i\right]}{n-1}$$

where $1[j\to i]$ equals 1 when classmate *j* nominated student *i* on the desk-sharing rejection item, and $n-1$ denotes the number of classmates who could have issued such a nomination. Higher scores on this index thus reflected greater perceived rejection within the classroom peer network.

Peer network measures showed moderate stability across the school year. The proportion of reciprocated friendships decreased slightly from *M* = 0.24 (*SD* = 0.14) at T1 to *M* = 0.18 (*SD* = 0.14) at T2, indicating some turnover in mutual peer ties over time. The proportion of exclusion nominations received remained stable, averaging *M* = 0.16 (*SD* = 0.10) at T1 and *M* = 0.16 (*SD* = 0.14) at T2. Missing data accounted for approximately 11% of nomination ties at T1 and 12% at T2.

#### Affect

Affective well-being was assessed using the 10-item Positive and Negative Affect Schedule (PANAS-10; Watson et al., 1988), which comprises five items measuring positive affect (e.g., enthusiastic, inspired, determined) and five items measuring negative affect (e.g., upset, nervous, distressed). Students were asked to rate how often each emotion had been experienced over the past few weeks on a Likert-type scale ranging from 1 (very slightly or not at all) to 5 (extremely). Missingness for the affect measures was modest, with 8% at T1 and 12% at T2. Internal consistency was acceptable to good for both subscales across both waves: positive affect, α =.82 (T1) and α =.83 (T2); negative affect, α =.81 (T1) and α =.80 (T2).

The expected two-factor structure of the PANAS-10 was evaluated through confirmatory factor analyses at each time point, estimated using the weighted least squares mean and variance adjusted (WLSMV) estimator appropriate for ordinal indicators. For positive affect, acceptable model fit was indicated at T1, χ^2^(5) = 97.68, p <.01, CFI =.98, TLI =.96, RMSEA =.09, 90% CI [.07,.10], SRMR =.03, and at T2, χ^2^(5) = 101.17, p <.01, CFI =.98, TLI =.96, RMSEA =.09, 90% CI [.08,.11], SRMR =.03. For negative affect, fit was similarly acceptable at T1, χ^2^(5) = 102.79, p <.01, CFI =.97, TLI =.95, RMSEA =.09, 90% CI [.08,.11], SRMR =.03, and at T2, χ^2^(5) = 108.86, p <.01, CFI =.97, TLI =.94, RMSEA =.09, 90% CI [.08,.11], SRMR =.03. Although chi-square tests were statistically significant at both time points, this was expected given the sensitivity of this statistic to large sample sizes; incremental and residual fit indices consistently indicated acceptable fit of the two-factor structure.

Longitudinal measurement invariance of the affect scales was subsequently examined through a sequence of configural, metric, and scalar models estimated in a multigroup framework with wave (T1 vs. T2) serving as the grouping variable. The configural model, in which no equality constraints were imposed across waves, demonstrated acceptable fit (CFI =.993, TLI =.991, RMSEA =.059, SRMR =.039), confirming that the same two-factor structure was maintained at both time points. Constraining factor loadings to equality across waves (metric model) produced no meaningful change in fit (ΔCFI =  −.000, ΔRMSEA =  −.003, ΔSRMR =  +.001), supporting metric invariance. The further imposition of equality constraints on item thresholds (scalar model) similarly resulted in no deterioration of fit (ΔCFI =  +.000, ΔRMSEA =  −.007, ΔSRMR =  −.000), with all indices remaining in the acceptable-to-excellent range (CFI =.993, TLI =.994, RMSEA =.049, SRMR =.040). Full scalar invariance was therefore supported, indicating that positive and negative affect were measured equivalently across waves and permitting meaningful comparisons of latent means and structural relations across time points in subsequent analyses.

Gender-based measurement invariance of the PANAS-10 scales was additionally evaluated at each wave using a multigroup confirmatory factor analytic framework. A sequence of configural, metric, and scalar models was estimated separately for T1 and T2. At both time points, the configural models demonstrated acceptable fit, indicating that the hypothesized two-factor structure was replicated across boys and girls (T1: CFI =.995, TLI =.993, RMSEA =.048, SRMR =.037; T2: CFI =.993, TLI =.991, RMSEA =.059, SRMR =.042). Imposing equality constraints on factor loadings resulted in negligible changes in fit indices (T1: ΔCFI =  −.001, ΔRMSEA =  +.001, ΔSRMR =  +.003; T2: ΔCFI =.000, ΔRMSEA =  −.001, ΔSRMR =  +.001), supporting metric invariance. Further constraining item thresholds to equality likewise produced no meaningful deterioration in model fit (T1: ΔCFI =.000, ΔRMSEA =  −.006, ΔSRMR =  −.002; T2: ΔCFI =.000, ΔRMSEA =  −.006, ΔSRMR =  −.001), with all indices remaining within recommended ranges. These results support full scalar invariance of the affect measures across gender at both waves, indicating that positive and negative affect were assessed equivalently for boys and girls and that subsequent gender comparisons can be interpreted as reflecting substantive differences rather than measurement artefacts.

#### Achievement

Academic achievement was assessed using standardized ability tests in mathematics and Czech language constructed within an Item Response Theory (IRT) framework. The use of IRT scaling enabled latent proficiency scores to be estimated on a common metric across measurement waves, thereby improving comparability of achievement levels over time and reducing measurement error associated with raw test scores.

The mathematics assessment consisted of 30 items and the Czech language assessment of 40 items. Both tests included a combination of multiple-choice, open-ended, and ordering tasks designed to capture a broad range of competencies within each domain. All students had same test items. The test content differed between the waves. All responses were coded dichotomously (1 = correct; 0 = incorrect or missing), following standard large-scale assessment procedures.

Ability estimates were derived using mixed-model IRT calibration. A three-parameter logistic (3PL) model was applied to multiple-choice items in order to account for pseudo-guessing, whereas a two-parameter logistic (2PL) model was used for open-ended and ordering items, for which guessing effects are theoretically less relevant. This modelling strategy allowed domain-specific proficiency to be estimated with an appropriate balance between flexibility and parsimony across heterogeneous item formats.

The resulting latent achievement scores were subsequently linearly transformed to a standardized scale (M ≈ 100, SD ≈ 15 at baseline) to facilitate interpretability and comparability across domains and waves. Model fit indices indicated excellent correspondence between the measurement models and the observed response patterns in both mathematics (CFI =.99, TLI =.99, RMSEA =.03, SRMR =.03) and Czech language (CFI =.99, TLI =.98, RMSEA =.02, SRMR =.02), supporting the construct validity of the estimated proficiency scores.

Descriptive analyses indicated moderate average improvement in both domains across the school year. Mean mathematics performance increased from M = 100.25 (SD = 19.69) at T1 to M = 106.10 (SD = 17.70) at T2, while Czech language performance rose from M = 100.41 (SD = 14.88) to M = 103.78 (SD = 15.61). The number of valid observations per achievement indicator ranged between 292 and 304 classrooms, with approximately 11–12% missing data across waves.

#### Demographic items

Demographic covariates included gender, ethnicity, and parental education, all assessed through student self-reports. Gender was coded as a binary variable boy/girl. Ethnicity was defined according to language use at home: students were classified as non-Czech if at least one parent regularly spoke a language other than Czech at home. Parental education was coded as a binary indicator distinguishing students with at least one parent holding a higher-education degree from those whose parents did not. These covariates were included as controls in all models to account for demographic variability in affective, social, and academic outcomes, as well as to test the moderating role of gender in the associations between peer relations, affect, and academic achievement.

### Analytical procedure

To address the research questions and test the hypotheses, a cross-lagged panel model (CLPM) using the *lavaan* package (Rosseel, 2012) in *R* (R Core Team, 2021) was estimated. The CLPM captures three types of associations: (a) cross-sectional paths, representing contemporaneous relationships among variables within each wave; (b) stability paths, capturing autoregressive effects that reflect the temporal consistency of constructs across waves; and (c) cross-lagged paths, which estimate directional influences of one construct on another over time, while controlling for prior levels.

A single integrated CLPM, incorporating peer relations (friendship and social exclusion), dimensions of affect (positive and negative), demographic covariates (gender, ethnicity, parental education), and interaction terms with gender as predictors, with affective and academic outcomes (mathematics and Czech achievement) as endogenous variables, was specified. This integrated specification was preferred over multigroup modeling because it enables moderation effects to be estimated directly while retaining the full analytic sample and maximizing statistical power. Moreover, modeling gender interactions within a single structural system allows differences in the strength of specific pathways to be examined without imposing the strong assumption that the overall model structure and measurement properties operate equivalently across groups.

No latent variables were included directly in the structural model due to its complexity and high parameterization. Instead, the structural-after-measurement (SAM) approach (Rosseel & Loh, 2024) was applied. SAM separates the measurement and structural components to improve estimation stability and computational efficiency. In the first step, a measurement model, including confirmatory factor analyses for the two affect dimensions and IRT-based models for mathematics and Czech achievement, was fitted. The measurement model validated the measurement properties and ensured adequate construct representation. In the second step, the factor scores extracted from these validated measurement models were entered as observed indicators in the CLPM to estimate the structural relations among peer relations, affect, and academic achievement.

Missing data were handled using full information maximum likelihood (FIML), which uses all available observed information under the assumption that data are missing at random (MAR) and is generally less biased and more efficient than listwise deletion (Baraldi & Enders, 2010). Because the MAR assumption cannot be tested directly, missingness was examined by comparing complete and incomplete cases on key observed study variables. Approximately 24.9% of participants had missing data on at least one variable included in the analytic model. Incomplete cases showed somewhat lower baseline positive affect, higher baseline negative affect, lower baseline mathematics and Czech language achievement, and a higher proportion of students with minority-language/ethnic background, whereas no meaningful differences were observed for gender or parental education. This pattern suggests that missingness was related to observed characteristics rather than being completely random, which is consistent with the use of FIML. Supplementary Material S1 provides detailed descriptive comparisons between complete and incomplete cases across all study variables.

## Results

**Fig. 1:**
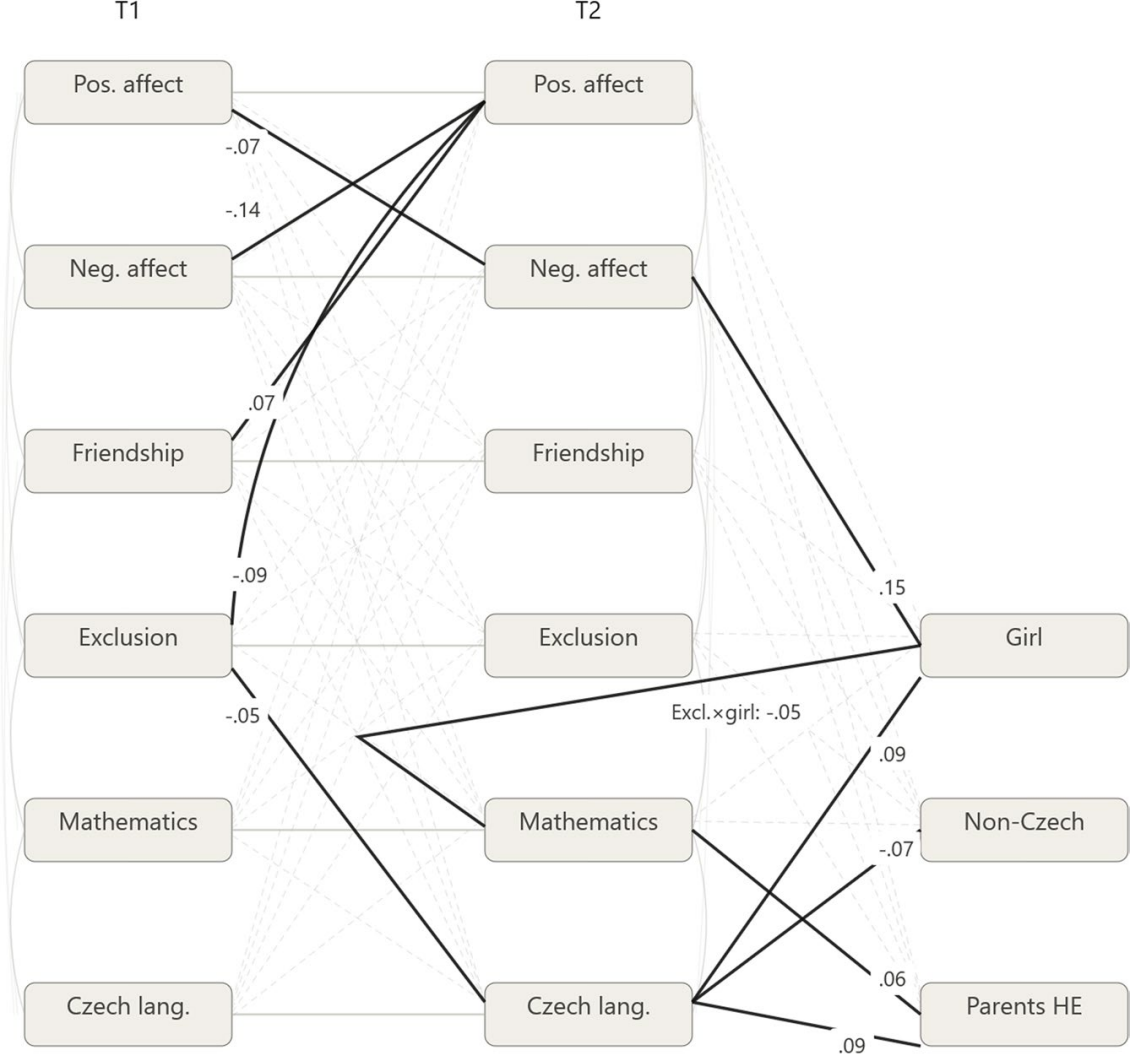
Model diagram with resulting paths and standardized regression coefficients for the significant paths of interest

Figure 1 displays the full cross-lagged panel model. Significant paths are shown as solid dark arrows with standardized coefficients; non-significant paths, stability coefficients, and residual covariances are represented as faded lines to reduce visual clutter while preserving the completeness of the model specification. Table 3 shows the corresponding CLPM results, focusing on the cross-lagged relations only, as these are the primary tests of temporal directionality. Supplementary Material S2 contains the rest of the CLPM estimates, including the autoregressive paths, covariances at T1 and T2, intercepts, and variances. The model demonstrated good fit to the data (CFI =.985, TLI =.905, RMSEA =.057, 90% CI [.051,.064], SRMR =.021).

**Table 3:** CLPM results

effect	*β*	*b*	*SE*	*p*	CI lower	CI upper
*predictors of positive affect*						
positive affect T2 ~ positive affect T1	.36	.37	.03	<.01	.31	.42
positive affect T2 ~ negative affect T1	-.14	-.14	.03	<.01	-.19	-.09
positive affect T2 ~ mathematics IRT T1	-.01	.00	.00	.67	.00	.00
positive affect T2 ~ Czech language IRT T1	.00	.00	.00	.99	.00	.00
positive affect T2 ~ friendship T1	.07	.34	.14	.02	.06	.62
positive affect T2 ~ exclusion T1	-.09	-.53	.15	<.01	-.83	-.23
positive affect T2 ~ friendship X girl T1	.00	-.02	.19	.91	-.40	.36
positive affect T2 ~ exclusion X girl T1	.01	.08	.24	.73	-.39	.55
positive affect T2 ~ girl	-.02	-.03	.04	.45	-.10	.05
positive affect T2 ~ non-Czech	-.02	-.07	.08	.39	-.22	.09
positive affect T2 ~ parents with HE	.02	.03	.04	.35	-.04	.10
*predictors of negative affect*						
negative affect T2 ~ positive affect T1	-.07	-.07	.02	.01	-.11	-.02
negative affect T2 ~ negative affect T1	.47	.47	.02	<.01	.43	.52
negative affect T2 ~ mathematics IRT T1	.02	.00	.00	.41	.00	.00
negative affect T2 ~ Czech language IRT T1	.04	.00	.00	.14	.00	.01
negative affect T2 ~ friendship T1	-.03	-.15	.13	.25	-.41	.11
negative affect T2 ~ exclusion T1	.05	.27	.14	.06	-.01	.54
negative affect T2 ~ friendship X girl T1	.00	.01	.18	.97	-.35	.37
negative affect T2 ~ exclusion X girl T1	-.02	-.15	.21	.48	-.57	.27
negative affect T2 ~ girl	.15	.28	.04	<.01	.21	.35
negative affect T2 ~ non-Czech	-.01	-.05	.07	.49	-.18	.08
negative affect T2 ~ parents with HE	.01	.02	.03	.48	-.04	.09
*predictors of mathematics achievement*						
mathematics IRT T2 ~ positive affect T1	.01	.11	.37	.77	-.62	.83
mathematics IRT T2 ~ negative affect T1	-.04	-.68	.36	.06	−1.39	.03
mathematics IRT T2 ~ mathematics IRT T1	.65	.57	.02	<.01	.54	.60
mathematics IRT T2 ~ friendship T1	.03	2.96	2.31	.20	−1.57	7.48
mathematics IRT T2 ~ exclusion T1	-.01	−1.09	2.29	.63	−5.58	3.40
mathematics IRT T2 ~ friendship X girl T1	.00	-.56	3.15	.86	−6.75	5.62
mathematics IRT T2 ~ exclusion X girl T1	-.05	−8.02	3.32	.02	−14.53	−1.50
mathematics IRT T2 ~ girl	-.02	-.78	.57	.17	−1.90	.34
mathematics IRT T2 ~ non-Czech	-.02	−1.59	1.02	.12	−3.59	.42
mathematics IRT T2 ~ parents with HE	.06	2.13	.58	<.01	.99	3.26
*predictors of Czech language achievement*						
Czech language IRT T2 ~ positive affect T1	.02	.41	.33	.21	-.23	1.04
Czech language IRT T2 ~ negative affect T1	-.01	-.21	.34	.54	-.87	.46
Czech language IRT T2 ~ Czech language IRT T1	.62	.64	.02	<.01	.60	.67
Czech language IRT T2 ~ friendship T1	.01	.75	1.88	.69	−2.93	4.42
Czech language IRT T2 ~ exclusion T1	-.05	−4.59	2.06	.03	−8.63	-.55
Czech language IRT T2 ~ friendship X girl T1	.00	-.05	2.54	.99	−5.02	4.93
Czech language IRT T2 ~ exclusion X girl T1	-.02	−2.31	3.13	.46	−8.44	3.82
Czech language IRT T2 ~ girl	.09	2.61	.50	<.01	1.63	3.60
Czech language IRT T2 ~ non-Czech	-.07	−4.15	1.05	<.01	−6.21	−2.10
Czech language IRT T2 ~ parents with HE	.09	2.80	.49	<.01	1.84	3.77

Regarding **H1** (peer relations influencing affect), the results clearly supported the hypotheses for positive affect, and provided mixed evidence for negative affect. As predicted, adolescents who reported stronger friendships at the beginning of the school year subsequently showed higher positive affect, whereas those who experienced greater peer exclusion reported lower positive affect. Both effects were statistically significant and consistent in direction with theoretical expectations, confirming **H1a** and **H1c**. In contrast, friendship did not significantly predict changes in negative affect over time, providing no support for **H1b**. Social exclusion, however, showed a marginal prospective association with increases in negative affect (95% CI [−.01,.54]), which may be interpreted as tentative evidence in support of **H1d**.

Turning to **H2** (peer relations influencing achievement), the results provided partial support. Overall, peer relations did not significantly predict later mathematics performance, providing no support for **H2c**. In contrast, exclusion had an overall significant negative effect on Czech language achievement, confirming **H2d**. Friendship did not predict academic outcomes in either domain, providing no support for **H2a** or **H2b**. These results underscore the detrimental role of exclusion rather than the protective role of friendship for academic development.

Concerning **H3** (affect influencing achievement), the findings did not support the expected prospective effects of positive or negative affect on subsequent academic performance. Neither dimension of affect significantly predicted later achievement in mathematics or Czech language once prior performance and other covariates were controlled. Although negative affect showed a small, marginally significant tendency to lower later mathematics scores (95% CI [−1.39,.03], providing tentative support for **H3c**. The rest of the hypothesized directional influence of affect on academic outcomes (**H3a**, **H3b**, **H3d**) was not confirmed.

Finally, with respect to **H4** (gender moderation), evidence was limited but theoretically meaningful. The interaction between exclusion and gender was significant in predicting mathematics achievement, indicating that girls who felt excluded early in the school year tended to perform worse in mathematics later on, whereas boys’ performance was unaffected. The results thus provide support for **H4d**. No gender moderation emerged for Czech language or for the links between peer relations and affect (**H4a**, **H4b**, **H4c**).

Beyond the hypothesized paths, several additional findings from the CLPM merit attention. Positive and negative affect showed significant negative cross-lagged links in both directions. Adolescents who reported higher positive affect at the start of the year subsequently experienced fewer negative emotions, and those who initially felt more negative reported lower positive affect later. This pattern indicates a dynamic inverse relationship between the two affective dimensions: rather than being entirely separate emotional systems, they tend to fluctuate in opposition across time. Among demographic covariates, girls reported higher negative affect overall and outperformed boys in Czech language, whereas no gender difference emerged in mathematics once peer and affective factors were included. Students of non-Czech origin tended to score lower in Czech language and showed a marginal trend toward lower mathematics performance, reflecting possible linguistic or integration-related disadvantages. In contrast, having at least one higher-educated parent consistently predicted higher achievement across both subjects, underscoring the enduring role of socioeconomic background in academic outcomes. On the other hand, reverse cross-lagged paths from prior academic achievement to subsequent affect were all null, indicating that academic performance was not associated with a detectable lagged influence on general affective well-being over the course of the school year.

In summary, the cross-lagged results demonstrate that peer relations exert a robust influence on adolescents’ positive emotional well-being, but only limited effects on negative affect and academic outcomes. Exclusion was detrimental, lowering both emotional well-being and Czech language achievement and showing a gender-specific negative effect on mathematics performance for girls. In contrast, positive affect and friendship did not translate into measurable academic gains over time. These findings suggest that the social costs of exclusion, rather than the benefits of friendship, play a more decisive role in shaping adolescents’ emotional and academic trajectories.

To assess the robustness of the findings, two sensitivity analyses were conducted. First, the model was refit without the gender interaction terms, retaining only main effects, to examine whether the key cross-lagged estimates were sensitive to the inclusion of the interaction specification. Second, the model was refit using listwise deletion rather than FIML to evaluate whether the handling of missing data influenced the conclusions. In both cases, the pattern of significant cross-lagged paths and the direction and magnitude of the standardized estimates were consistent with those obtained in the primary analysis. Full results of both sensitivity analyses, including the key path estimates, are reported in Supplementary Material S3.

## Discussion

The present study extends existing research on the interplay between peer relations, affective well-being, and academic achievement in adolescence by applying a two-wave cross-lagged panel model to a nationally stratified Czech sample. The findings largely corroborate prior work emphasizing the importance of peer connectedness for adolescents’ socio-emotional functioning (Chiu et al., 2021; Liu et al., 2024; Tikkanen et al., 2024; Wentzel et al., 2021), but they also refine this literature by revealing that these effects are stronger for positive affect than for academic performance. Consistent with the self-system model of motivational development (Connell & Wellborn, 1991) and self-determination theory (Deci et al., 2017; Germani & Vespasiani, 2023), adolescents who felt accepted and connected to their peers reported more positive affect, whereas those who felt excluded experienced declines in well-being. These patterns align with prior longitudinal findings showing that supportive peer relationships promote emotional adjustment (Kiuru et al., 2020; Shao et al., 2024) and eudaimonic well-being (Ju et al., 2025). However, the absence of significant cross-lagged effects on negative affect suggests that peer relations may operate primarily through enhancing positive emotional resources, rather than directly reducing negative emotional states. This asymmetry is consistent with evidence that positive and negative affect are not merely opposite poles of a continuum but functionally distinct processes, with positive affect reflecting engagement and motivation, and negative affect reflecting reactivity or withdrawal.

The finding that peer exclusion predicted lower Czech language achievement for all students, and lower mathematics achievement specifically among girls, provides an important refinement to prior evidence on gendered academic vulnerability (John et al., 2023; Robnett & Leaper, 2013; Rose & Rudolph, 2006). These results can be read alongside the recent findings of Rémeau et al. (2025), who examined reciprocal relations between subjective school well-being, numeracy, and literacy across Grades 5 to 8, using similar design. They found that academic achievement — particularly numeracy — predicted subsequent school well-being, but that this effect was concentrated among girls and low-SES students. Notably, the direction of effect in Rémeau et al. ran from achievement to well-being rather than the reverse, which contrasts with the peer-to-achievement pathway identified in the present study. Two differences in design may account for this divergence. The outcome constructs differ meaningfully: where Rémeau et al. assessed a multidimensional school well-being construct encompassing satisfaction, peer relations, and teacher–student relationships, the present study used the PANAS to capture general positive and negative affect. Achievement may shape contextualized, school-specific well-being more readily than broad hedonic affect, given how proximally the former is connected to the academic domain. The time lag also differs substantially — three academic years versus one. Taken together, the two studies suggest that the relationship between social-emotional experience and academic outcomes is not uniformly bidirectional, but rather asymmetric and moderated by sex and social context: whereas prior achievement shapes subsequent well-being in ways that disproportionately affect girls, the present study demonstrates that social experiences such as peer exclusion can in turn constrain academic performance, again with differential effects by gender.

One plausible explanation is that social belonging and perceived inclusion are more tightly intertwined with girls’ academic self-concept, particularly in male-stereotyped domains such as mathematics. Social exclusion may thus undermine girls’ sense of competence and belonging in mathematics, amplifying stereotype threat and reducing engagement. Girls who feel isolated in mathematics-related contexts may internalize the message that they do not “fit” in achievement-oriented peer groups, leading to lower confidence and diminished persistence. In contrast, the absence of a comparable effect for Czech language—a domain perceived as more gender-neutral—suggests that the emotional cost of exclusion is domain-specific, emerging when social and academic identities intersect with cultural stereotypes. Future research should explore these mechanisms more directly, integrating measures of perceived belonging, stereotype threat, and academic identity to clarify how social and cognitive pathways jointly mediate the link between peer exclusion and performance.

Interestingly, neither friendship nor exclusion predicted subsequent negative affect, which contrasts with the expected pattern and warrants careful interpretation. One possible explanation concerns limited subjective awareness of social exclusion. In this study, exclusion was operationalized as the in-degree of negative nominations — how many classmates reported not wanting to share a desk with a given student. However, adolescents may not be fully aware of the extent to which they are excluded by peers, particularly when exclusion occurs subtly or indirectly. Research on meta-accuracy in peer relations has shown that children and adolescents frequently misperceive their standing within the peer group, with discrepancies between actual sociometric status and self-perceived acceptance being common (Cillessen & Bellmore, 2003). Without awareness of being excluded, the experience may not translate into consciously felt negative emotions. This interpretation is further supported by daily diary research demonstrating that the affective consequences of peer harassment are most pronounced on days when victimization is directly and personally experienced — suggesting that proximity and salience of the social experience are necessary conditions for affective responses to emerge (Nishina & Juvonen, 2005). Another explanation concerns measurement specificity: the PANAS negative affect items capture general distress and irritability rather than socially oriented emotions such as loneliness or rejection sensitivity (Watson et al., 1988). Consequently, the affective consequences of peer exclusion may be underestimated when assessed through a general negative affect scale rather than one focused on social emotions.

Contrary to expectations, the hypotheses predicting effects of affect on later achievement were not supported. Neither positive nor negative affect predicted subsequent performance once prior achievement and other covariates were controlled for. This lack of effect may reflect the strong stability of academic achievement across the school year, leaving little residual variance for affect to explain. It may also suggest that the influence of affective states on learning is more proximal and transient than can be captured in a two-wave annual design. Daily or momentary fluctuations in affect may influence study effort or engagement in the short term, but such effects could average out over months, particularly in highly structured educational contexts. Furthermore, affect may affect achievement indirectly through behavioral engagement or motivation—mechanisms not directly modeled here. Previous studies that have found affect–achievement links (e.g., Schnell et al., 2025; Shao et al., 2024) often included such mediators or employed shorter temporal intervals. The absence of significant effects in the present study thus highlights the importance of examining affective processes at multiple timescales, combining longitudinal and experience-sampling approaches to capture both enduring and momentary components of emotional functioning in academic life.

Taken together, these findings underscore the central role of social belonging in adolescents’ well-being and learning, but they also reveal important boundary conditions. Friendship and inclusion primarily enhance positive emotional adjustment, while exclusion exerts domain- and gender-specific costs on academic outcomes. The results suggest that fostering socially inclusive classroom climates may be especially critical for girls in mathematics, where exclusion can undermine both emotional well-being and academic confidence. Future studies using multilevel, multi-wave designs will be crucial to clarify how classroom norms, gender stereotypes, and affective dynamics jointly shape adolescents’ developmental trajectories in school.

### Limitations

Several limitations should be considered when interpreting the findings.

First, the structural model was estimated at the individual level and did not incorporate a multilevel specification due to convergence constraints and model complexity. As classroom contexts represent important shared social environments, the omission of classroom-level random effects may have resulted in slightly underestimated standard errors and limited the ability to capture contextual influences on adolescents’ adjustment (Eccles & Roeser, 2011; Wentzel et al., 2021).

Second, the study relied on two measurement waves, which restricted the modelling of longer-term developmental processes and prevented the estimation of random-intercept cross-lagged panel models. Consequently, the reported cross-lagged paths should be interpreted as population-average prospective associations rather than within-person causal effects (Hamaker et al., 2015).

Third, although peer embeddedness was operationalized using network-based indicators, the design did not allow the separation of social selection from peer influence processes. Previous research suggests that both mechanisms contribute to developmental similarity among adolescents (Bukowski et al., 2011; Kiuru et al., 2020), and future studies employing stochastic network models could clarify these dynamics.

Fourth, academic achievement was assessed using externally scaled IRT ability scores. Because item-level response data were not accessible, differential item functioning across demographic subgroups could not be formally examined, which may limit the interpretation of group differences.

Finally, the study was conducted within a single national educational context characterized by relatively stable classroom groupings and early academic differentiation. Institutional and cultural features of the Czech school system may therefore shape the strength and form of associations between peer relations, emotional well-being, and achievement, potentially limiting generalizability to other educational settings (Wentzel et al., 2021; Yu et al., 2023).

### Implications

This study yields several important implications for future research. First, the findings highlight the need for longer-term, multi-wave designs that can distinguish between within-person and between-person processes, allowing for a clearer understanding of how peer dynamics and affect evolve over time. Future studies should also adopt multilevel frameworks to capture classroom- and school-level influences, as peer relations are inherently contextual and shaped by group norms. Moreover, it will be essential to disentangle subject- and gender-specific effects more precisely—particularly to understand why girls appear more vulnerable to the negative academic consequences of social exclusion in mathematics. Further studies could help uncover the social, emotional, and identity-based mechanisms underlying this pattern, such as stereotype threat, perceived competence, or the emotional meaning attached to peer belonging in male-dominated domains. Finally, comparative studies across educational systems may clarify whether these gendered effects are culturally contingent or reflect broader developmental regularities in adolescence.

## Supplementary Information

Below is the link to the electronic supplementary material.

Supplementary file1 (DOCX 48 KB)

## Data Availability

The data that support the findings of this study are openly available in ČSDA at 10.14473/CSDA/I17GF8.
