# Supplementary material for: Friendship fosters well-being, exclusion may hurt girls’ mathematics: a two-wave cross-lagged panel model of peer relations, affect, and achievement
Source: Eur J Psychol Educ. 2026 May 30;41(2):79. doi: 10.1007/s10212-026-01138-6 (PMC13222188; doi:10.1007/s10212-026-01138-6)
Supplement: Supplementary file 1 — Supplementary file1 (DOCX 48 KB) [file 10212_2026_1138_MOESM1_ESM.docx]

**Supplementary Material for *Friendship Fosters Well-Being, Exclusion May Hurt Girls’ Mathematics: A Two-Wave Cross-Lagged Panel Model of Peer Relations, Affect, and Achievement***

**S1: Missingness Analysis**

| variable | M incomplete | SD incomplete | M complete | SD complete | test used | test statistics | *p* |
| --- | --- | --- | --- | --- | --- | --- | --- |
| positive affect T1 | -0.12 | 0.97 | 0.03 | 0.92 | t-test | -2.98 | <0.00 |
| negative affect T1 | 0.09 | 0.97 | -0.02 | 0.91 | t-test | 2.13 | 0.03 |
| mathematics IRT T1 | 97.40 | 18.78 | 100.96 | 19.80 | t-test | -3.27 | <0.00 |
| Czech language IRT T1 | 98.40 | 15.03 | 100.57 | 14.84 | t-test | -2.48 | 0.01 |
| friendship T1 | 0.15 | 0.17 | 0.27 | 0.18 | t-test | -15.87 | <0.00 |
| exclusion T1 | 0.16 | 0.17 | 0.16 | 0.16 | t-test | 0.22 | 0.83 |
| gender | 0.53 |  | 0.50 |  | χ2 | 1.29 | 0.26 |
| ethnicity | 0.12 |  | 0.07 |  | χ2 | 6.57 | 0.01 |
| parental education | 0.43 |  | 0.43 |  | χ2 | 0.00 | 0.98 |

**S2: Full CLPM Results**

| effect | *b* | *SE* | *p* | CI lower | CI upper |
| --- | --- | --- | --- | --- | --- |
| *cross-lagged paths* |  |  |  |  |  |
| Czech language IRT T2 ~ ethnicity | -4.15 | 1.05 | <.01 | -6.21 | -2.1 |
| Czech language IRT T2 ~ exclusion T1 | -4.59 | 2.06 | .03 | -8.63 | -.55 |
| Czech language IRT T2 ~ exclusion X girl T1 | -2.31 | 3.13 | .46 | -8.44 | 3.82 |
| Czech language IRT T2 ~ friendship T1 | .75 | 1.88 | .69 | -2.93 | 4.42 |
| Czech language IRT T2 ~ friendship X girl T1 | -.05 | 2.54 | .99 | -5.02 | 4.93 |
| Czech language IRT T2 ~ gender | 2.61 | .5 | <.01 | 1.63 | 3.6 |
| Czech language IRT T2 ~ negative affect T1 | -.21 | .34 | .54 | -.87 | .46 |
| Czech language IRT T2 ~ parental education | 2.8 | .49 | <.01 | 1.84 | 3.77 |
| Czech language IRT T2 ~ positive affect T1 | .41 | .33 | .21 | -.23 | 1.04 |
| math IRT T2 ~ ethnicity | -1.59 | 1.02 | .12 | -3.59 | .42 |
| math IRT T2 ~ exclusion T1 | -1.09 | 2.29 | .63 | -5.58 | 3.4 |
| math IRT T2 ~ exclusion X girl T1 | -8.02 | 3.32 | .02 | -14.53 | -1.5 |
| math IRT T2 ~ friendship T1 | 2.96 | 2.31 | .20 | -1.57 | 7.48 |
| math IRT T2 ~ friendship X girl T1 | -.56 | 3.15 | .86 | -6.75 | 5.62 |
| math IRT T2 ~ gender | -.78 | .57 | .17 | -1.9 | .34 |
| math IRT T2 ~ negative affect T1 | -.68 | .36 | .06 | -1.39 | .03 |
| math IRT T2 ~ parental education | 2.13 | .58 | <.01 | .99 | 3.26 |
| math IRT T2 ~ positive affect T1 | .11 | .37 | .77 | -.62 | .83 |
| negative affect T2 ~ Czech language IRT T1 | .00 | .00 | .14 | .00 | .01 |
| negative affect T2 ~ ethnicity | -.05 | .07 | .49 | -.18 | .08 |
| negative affect T2 ~ exclusion T1 | .27 | .14 | .06 | -.01 | .54 |
| negative affect T2 ~ exclusion X girl T1 | -.15 | .21 | .48 | -.57 | .27 |
| negative affect T2 ~ friendship T1 | -.15 | .13 | .25 | -.41 | .11 |
| negative affect T2 ~ friendship X girl T1 | .01 | .18 | .97 | -.35 | .37 |
| negative affect T2 ~ gender | .28 | .04 | <.01 | .21 | .35 |
| negative affect T2 ~ math IRT T1 | .00 | .00 | .41 | .00 | .00 |
| negative affect T2 ~ parental education | .02 | .03 | .48 | -.04 | .09 |
| negative affect T2 ~ positive affect T1 | -.07 | .02 | <.01 | -.11 | -.02 |
| positive affect T2 ~ Czech language IRT T1 | .00 | .00 | .99 | .00 | .00 |
| positive affect T2 ~ ethnicity | -.07 | .08 | .39 | -.22 | .09 |
| positive affect T2 ~ exclusion T1 | -.53 | .15 | <.01 | -.83 | -.23 |
| positive affect T2 ~ exclusion X girl T1 | .08 | .24 | .73 | -.39 | .55 |
| positive affect T2 ~ friendship T1 | .34 | .14 | .02 | .06 | .62 |
| positive affect T2 ~ friendship X girl T1 | -.02 | .19 | .91 | -.4 | .36 |
| positive affect T2 ~ gender | -.03 | .04 | .45 | -.1 | .05 |
| positive affect T2 ~ math IRT T1 | .00 | .00 | .67 | .00 | .00 |
| positive affect T2 ~ negative affect T1 | -.14 | .03 | <.01 | -.19 | -.09 |
| positive affect T2 ~ parental education | .03 | .04 | .35 | -.04 | .10 |
| *autoregressive paths* |  |  |  |  |  |
| Czech language IRT T2 ~ Czech language IRT T1 | .64 | .02 | <.01 | .6 | .67 |
| exclusion T2 ~ exclusion T1 | .44 | .02 | <.01 | .4 | .48 |
| friendship T2 ~ friendship T1 | .32 | .02 | <.01 | .28 | .36 |
| math IRT T2 ~ math IRT T1 | .57 | .02 | <.01 | .54 | .60 |
| negative affect T2 ~ negative affect T1 | .47 | .02 | <.01 | .43 | .52 |
| positive affect T2 ~ positive affect T1 | .37 | .03 | <.01 | .31 | .42 |
| *T1 covariances and predictions* |  |  |  |  |  |
| Czech language IRT T1 ~ ethnicity | -9.63 | 1.17 | <.01 | -11.92 | -7.33 |
| Czech language IRT T1 ~ gender | 4.91 | .62 | <.01 | 3.71 | 6.12 |
| Czech language IRT T1 ~ parental education | 5.2 | .63 | <.01 | 3.97 | 6.43 |
| Czech language IRT T1 ~~ Czech language IRT T1 | 202.69 | 5.92 | <.01 | 191.09 | 214.29 |
| Czech language IRT T1 ~~ exclusion X girl T1 | -.25 | .03 | <.01 | -.32 | -.19 |
| Czech language IRT T1 ~~ friendship X girl T1 | .12 | .04 | <.01 | .05 | .20 |
| exclusion T1 ~ ethnicity | .03 | .01 | .01 | .01 | .06 |
| exclusion T1 ~ gender | -.04 | .01 | <.01 | -.05 | -.03 |
| exclusion T1 ~ parental education | -.02 | .01 | .01 | -.03 | .00 |
| exclusion T1 ~~ Czech language IRT T1 | -.45 | .05 | <.01 | -.55 | -.35 |
| exclusion T1 ~~ exclusion T1 | .03 | .00 | <.01 | .02 | .03 |
| exclusion T1 ~~ exclusion X girl T1 | .01 | .00 | <.01 | .01 | .01 |
| exclusion T1 ~~ friendship X girl T1 | .00 | .00 | <.01 | .00 | .00 |
| exclusion T1 ~~ math IRT T1 | -.58 | .07 | <.01 | -.71 | -.45 |
| exclusion X girl T1 ~~ exclusion X girl T1 | .01 | .00 | <.01 | .01 | .01 |
| friendship T1 ~ ethnicity | -.03 | .01 | .03 | -.06 | .00 |
| friendship T1 ~ gender | .00 | .01 | .99 | -.02 | .01 |
| friendship T1 ~ parental education | .01 | .01 | .25 | -.01 | .02 |
| friendship T1 ~~ Czech language IRT T1 | .24 | .05 | <.01 | .14 | .35 |
| friendship T1 ~~ exclusion T1 | -.01 | .00 | <.01 | -.01 | .00 |
| friendship T1 ~~ exclusion X girl T1 | .00 | .00 | <.01 | .00 | .00 |
| friendship T1 ~~ friendship T1 | .03 | .00 | <.01 | .03 | .04 |
| friendship T1 ~~ friendship X girl T1 | .02 | .00 | <.01 | .02 | .02 |
| friendship T1 ~~ math IRT T1 | .25 | .07 | <.01 | .12 | .39 |
| friendship X girl T1 ~~ exclusion X girl T1 | .00 | .00 | <.01 | .00 | .00 |
| friendship X girl T1 ~~ friendship X girl T1 | .02 | .00 | <.01 | .02 | .02 |
| math IRT T1 ~ ethnicity | -7.83 | 1.51 | <.01 | -1.79 | -4.88 |
| math IRT T1 ~ gender | -3.12 | .83 | <.01 | -4.75 | -1.49 |
| math IRT T1 ~ parental education | 7.31 | .85 | <.01 | 5.65 | 8.97 |
| math IRT T1 ~~ Czech language IRT T1 | 174.14 | 6.69 | <.01 | 161.03 | 187.25 |
| math IRT T1 ~~ exclusion X girl T1 | -.26 | .04 | <.01 | -.34 | -.18 |
| math IRT T1 ~~ friendship X girl T1 | .14 | .05 | <.01 | .04 | .23 |
| math IRT T1 ~~ math IRT T1 | 369.5 | 11.08 | <.01 | 347.79 | 391.21 |
| negative affect T1 ~ ethnicity | .03 | .07 | .68 | -.11 | .17 |
| negative affect T1 ~ gender | .43 | .04 | <.01 | .35 | .50 |
| negative affect T1 ~ parental education | -.03 | .04 | .41 | -.11 | .04 |
| negative affect T1 ~~ Czech language IRT T1 | -.61 | .28 | .03 | -1.15 | -.07 |
| negative affect T1 ~~ exclusion T1 | .01 | .00 | <.01 | .01 | .02 |
| negative affect T1 ~~ exclusion X girl T1 | .01 | .00 | <.01 | .00 | .01 |
| negative affect T1 ~~ friendship T1 | -.02 | .00 | <.01 | -.03 | -.01 |
| negative affect T1 ~~ friendship X girl T1 | -.01 | .00 | <.01 | -.02 | -.01 |
| negative affect T1 ~~ math IRT T1 | -1.29 | .37 | <.01 | -2.01 | -.57 |
| negative affect T1 ~~ negative affect T1 | .8 | .02 | <.01 | .76 | .84 |
| positive affect T1 ~ ethnicity | .00 | .08 | 1.00 | -.16 | .16 |
| positive affect T1 ~ gender | -.11 | .04 | .01 | -.19 | -.03 |
| positive affect T1 ~ parental education | .06 | .04 | .14 | -.02 | .14 |
| positive affect T1 ~~ Czech language IRT T1 | 2.04 | .29 | <.01 | 1.48 | 2.61 |
| positive affect T1 ~~ exclusion T1 | -.02 | 0 | <.01 | -.03 | -.02 |
| positive affect T1 ~~ exclusion X girl T1 | -.01 | 0 | <.01 | -.02 | -.01 |
| positive affect T1 ~~ friendship T1 | .03 | 0 | <.01 | .03 | .04 |
| positive affect T1 ~~ friendship X girl T1 | .02 | 0 | <.01 | .01 | .02 |
| positive affect T1 ~~ math IRT T1 | 2.52 | .37 | <.01 | 1.8 | 3.25 |
| positive affect T1 ~~ negative affect T1 | -.5 | .02 | <.01 | -.55 | -.46 |
| positive affect T1 ~~ positive affect T1 | .86 | .03 | <.01 | .81 | .91 |
| *T2 covariances* |  |  |  |  |  |
| Czech language IRT T2 ~~ exclusion T2 | -.03 | .03 | .30 | -.09 | .03 |
| Czech language IRT T2 ~~ friendship T2 | 0 | .03 | .92 | -.06 | .05 |
| friendship T2 ~~ exclusion T2 | 0 | 0 | .03 | 0 | 0 |
| math IRT T2 ~~ Czech language IRT T2 | 39.06 | 3.99 | <.01 | 31.23 | 46.88 |
| math IRT T2 ~~ exclusion T2 | -.07 | .03 | .02 | -.13 | -.01 |
| math IRT T2 ~~ friendship T2 | -.06 | .03 | .10 | -.12 | .01 |
| negative affect T2 ~~ Czech language IRT T2 | -.5 | .19 | .01 | -.87 | -.13 |
| negative affect T2 ~~ exclusion T2 | 0 | 0 | .17 | 0 | .01 |
| negative affect T2 ~~ friendship T2 | 0 | 0 | .10 | -.01 | 0 |
| negative affect T2 ~~ math IRT T2 | -.83 | .22 | <.01 | -1.27 | -.4 |
| positive affect T2 ~~ Czech language IRT T2 | .88 | .21 | <.01 | .47 | 1.3 |
| positive affect T2 ~~ exclusion T2 | 0 | 0 | .16 | -.01 | 0 |
| positive affect T2 ~~ friendship T2 | .01 | 0 | .02 | 0 | .01 |
| positive affect T2 ~~ math IRT T2 | .61 | .23 | .01 | .16 | 1.06 |
| positive affect T2 ~~ negative affect T2 | -.36 | .02 | <.01 | -.39 | -.32 |
| demographic variables covariances |  |  |  |  |  |
| ethnicity ~~ parental education | .01 | 0 | .01 | 0 | .01 |
| gender ~~ ethnicity | 0 | 0 | .52 | -.01 | 0 |
| gender ~~ parental education | .01 | .01 | .22 | 0 | .02 |
| *variances* |  |  |  |  |  |
| Czech language IRT T2 ~~ Czech language IRT T2 | 121.66 | 4.63 | <.01 | 112.59 | 13.74 |
| ethnicity ~~ ethnicity | .07 | 0 | <.01 | .06 | .08 |
| exclusion T2 ~~ exclusion T2 | .01 | 0 | <.01 | .01 | .01 |
| friendship T2 ~~ friendship T2 | .02 | 0 | <.01 | .02 | .02 |
| gender ~~ gender | .25 | 0 | <.01 | .25 | .25 |
| math IRT T2 ~~ math IRT T2 | 159.39 | 5.96 | <.01 | 147.72 | 171.07 |
| negative affect T2 ~~ negative affect T2 | .57 | .02 | <.01 | .53 | .6 |
| parental education ~~ parental education | .25 | 0 | <.01 | .24 | .25 |
| positive affect T2 ~~ positive affect T2 | .66 | .02 | <.01 | .62 | .71 |

**S3: Sensitivity Analysis – Comparison of Key Paths Between Original, No Interaction, and Listwise Deletion Models**

|  | original | | | no interactions | | | listwise deletion | | |
| --- | --- | --- | --- | --- | --- | --- | --- | --- | --- |
| effect | ***b*** | ***SE*** | ***p*** | ***b*** | ***SE*** | ***p*** | ***b*** | ***SE*** | ***p*** |
| positive affect T2 ~ positive affect T1 | .37 | .03 | <.01 | .37 | .03 | <.01 | .37 | .03 | <.01 |
| positive affect T2 ~ negative affect T1 | -.14 | .03 | <.01 | -.14 | .03 | <.01 | -.14 | .03 | <.01 |
| positive affect T2 ~ friendship T1 | .34 | .14 | .02 | .33 | .10 | <.01 | .40 | .16 | .01 |
| positive affect T2 ~ exclusion T1 | -.53 | .15 | <.01 | -.50 | .12 | <.01 | -.52 | .17 | <.01 |
| negative affect T2 ~ positive affect T1 | -.07 | .02 | .01 | -.07 | .02 | .01 | -.07 | .02 | <.01 |
| negative affect T2 ~ negative affect T1 | .47 | .02 | <.01 | .47 | .02 | <.01 | .48 | .02 | <.01 |
| negative affect T2 ~ friendship T1 | -.15 | .13 | .25 | -.14 | .09 | .12 | -.15 | .14 | .30 |
| negative affect T2 ~ exclusion T1 | .27 | .14 | .06 | .21 | .11 | .05 | .34 | .15 | .02 |
| mathematics IRT T2 ~ positive affect T1 | .11 | .37 | .77 | .13 | .37 | .72 | .47 | .40 | .24 |
| mathematics IRT T2 ~ negative affect T1 | -.68 | .36 | .06 | -.67 | .36 | .06 | -.32 | .39 | .40 |
| mathematics IRT T2 ~ exclusion X girl T1 | -8.02 | 3.32 | .02 |  |  |  | -8.75 | 3.51 | .01 |
| Czech language IRT T2 ~ positive affect T1 | .41 | .33 | .21 | .42 | .33 | .20 | .58 | .34 | .09 |
| Czech language IRT T2 ~ negative affect T1 | -.21 | .34 | .54 | -.20 | .34 | .55 | .01 | .35 | .99 |
| Czech language IRT T2 ~ exclusion T1 | -4.59 | 2.06 | .03 | -5.53 | 1.56 | <.01 | -5.18 | 2.21 | .02 |
